# Supplementary material for: Optimization strategies for conservation of traditional dwellings in Hongcun Village, China, based on decay phenomena analysis
Source: PLoS One. 2022 Nov 7;17(11):e0276306. doi: 10.1371/journal.pone.0276306 (PMC9639817; doi:10.1371/journal.pone.0276306)
Supplement: Attachment — Submitted filename: Review-A-3.pdf [file pone.0276306.s002.pdf]

This paper describes the decay phenomena of and conservation practices applied to traditional dwellings in hongcun village, china.

Despite the good text, this article needs to address the following concerns:

1) The research works that were performed in this field in recent years (after 2016) are not mentioned. Please cite more references.

2) Please provide an overview of existing problems and the solutions and suggestions needed to address them in form of a flowchart.

3) Lack of a general "Conclusion" section after which suggestions are made.
