# Supplementary material for: Optimization strategies for conservation of traditional dwellings in Hongcun Village, China, based on decay phenomena analysis
Source: PLoS One. 2022 Nov 7;17(11):e0276306. doi: 10.1371/journal.pone.0276306 (PMC9639817; doi:10.1371/journal.pone.0276306)
Supplement: Attachment — Submitted filename: PONE-D-21-22843R1-Response to Reviewers.doc [file pone.0276306.s001.doc]

Dear editors,

On behalf of my co-authors, we thank you very much for giving us an opportunity to revise our manuscript, we appreciate very much for the positive and constructive comments and suggestions on our manuscript entitled “**On the Subject of the Decay Phenomena of and Conservation Practices Applied to Traditional Dwellings in Hongcun Village, China**” (Manuscript Number: PONE-D-21-22843).

Those comments are all valuable and very helpful for revising and improving our paper, as well as the important guiding significance to our researches. We have studied comments carefully and have made correction which we hope meet with approval. Revised portion are marked in the manuscript. The main corrections in the manuscript and the responds to the editors’ comments are as flowing:

1. Please ensure the manuscript meets PLOS ONE's style requirements.

***Response:*** The manuscript has been revised in accordance with the PLOS ONE's style requirements, including the manuscript, title, author and affiliations.

2. Please provide additional information regarding the specimens used in your study.

***Response:*** In the part “Methods”, the “No permits were required for the described study, which complied with all relevant regulations” is added.

3. Please complete your Competing Interests

***Response:*** “NO authors have competing interests” is indicated in the section of “Declarations of Competing Interest”.

4. The funding information

***Response:*** We have remove the funding information from the manuscript. And also, we would like to add the funding information in the Funding Statement section of the online submission form, as well as in the cover letter with the amended statements “This paper is funded from the CUMT Fundamental Research Funds for the Central Universities (No.2020SK17)”.

5. Data Availability statement

***Response:*** In the manuscript and the cover letter, the “Data Availability statement” is added with the text “All data, models, and code generated or used during the study appear in the submitted article”.

6. ORCID iD

***Response:*** The corresponding author has created the new ORCID iD, which is 0000-0002-6178-916X, and also updated the information in the Editorial Manager.

7. Figure copyright

***Response:*** The figure 1 has been supplied with a replacement figure that complies with the CC BY 4.0 license, and also specify in the figure caption text when a figure is similar but not identical to the original image.

8. Reference list

***Response:*** The reference list has been reviewed and ensured that it is complete and correct.

9. Additional Editor Comments

***Response:*** The contents related to the comments are added in the manuscrpt, including the materials properties used in the region's traditional constructions, the information for traditional constructions in terms of conservation, retrofitting, and any other maintaining and diagnosing process, and the specifice source of problems in the Hongcun traditional dwellings.

We would like to express our great appreciation to you for comments on our paper. Looking forward to hearing from you.

Thank you and best regards.

Yours sincerely,

Corresponding author:

Tingshen Li

litingshen1988@163.com
