# Supplementary material for: Optimization strategies for conservation of traditional dwellings in Hongcun Village, China, based on decay phenomena analysis
Source: PLoS One. 2022 Nov 7;17(11):e0276306. doi: 10.1371/journal.pone.0276306 (PMC9639817; doi:10.1371/journal.pone.0276306)
Supplement: Attachment — Submitted filename: PONE-D-21-22843R1-Response to Reviewers.doc [file pone.0276306.s003.doc]

Dear editors,

On behalf of my coauthors, we thank you very much for giving us an opportunity to revise our manuscript. We appreciate the positive and constructive comments and suggestions on our manuscript ***Optimization Strategies for Conservation of Traditional Dwellings in Hongcun Village, China, Based on Decay Phenomena Analysis*** (Number: PONE-D-21-22843).

First, I would like to mention that Tingshen Li’s affiliation has changed to ***College of Environmental Engineering, Xuzhou University of Technology, Xuzhou 221018, China,*** and the corresponding author has been changed to Prof. Minghao Zhang. At the same time, the funding source has also changed to the ***Xuzhou Science and Technology Plan Project (No. KC21305)***. We apologize for any inconvenience to the journal.

The comments provided were all valuable and very helpful for revising and improving our paper and providing important guidance to our research. We have studied the comments carefully and have made corrections that we hope will meet with your approval. The revised portions are marked in the manuscript. The main corrections in the manuscript and the responses to the editors’ comments are as follows:

**Reviewer 1-1**. The title is not clear and needs to be replaced by a more appropriate.

***Response:*** After comprehensively analyzing the full research, we revised the title of the paper to " Optimization Strategies for Conservation of Traditional Dwellings in Hongcun Village, China, Based on Decay Phenomena Analysis" in order to indicate the emphasis of this study.

**Reviewer 1-2.** Abstract should be re-written and reflect the whole performed study clearly.

***Response:*** The abstract has been completely rewritten, and the whole research process is clearly introduced according to the logical structure of the *Material and Decay Investigation*, the *Problematic Conservation Cases Analysis*, and the *Conservation Measures Optimization Strategies*.

**Reviewer 1-3-4.** The whole introduction should be re-written completely. Only a few papers were referenced in the introduction section without talking about their results deeply. The exact evaluation should be discussed in Introduction and also the previous assessed methods should be presented. In this area different studies have been done that necessary to present in Introduction as solution

***Response:*** The introduction was completely rewritten by adding the results of many previous studies regarding building materials, decay phenomena, and conservation measures for Hongcun traditional dwellings. Additionally, the **Research Significance and Methods** section was added to elaborate and summarize the deficiencies of previous research and to discuss the specific research methods and procedures of this study.

**Reviewer 1-5.** Please avoid writing Chinese in English paper

***Response:*** All Chinese characters in the article have been deleted.

**Reviewer 1-6.** The conclusion section should be added to summarize all main obtained results

***Response:*** The Conclusion section has been added to summarize all the main results in the three aspects of the *Material and Decay Investigation*, the *Problematic Conservation Cases Analysis*, and the *Conservation Measures Optimization Strategies*.

**Reviewer 1-7.** Divide different structures and present their information step by step: 1- location, 2- materials, 3- application, 4- problem and finally 5- your suggested solution

***Response:*** The overall structure of this study has been reclassified as the general introduction (including the general information about and location of Hongcun), the survey and summary of the main Hongcun building materials, the investigation and analysis of the existing decay phenomena, the problematic conservation cases and finally the strategies for optimizing conservation practices. The revised structure of this paper has a clearer overall logic.

**Reviewer 2-1.** The research works that were performed in this field in recent years (after 2016) are not mentioned. Please cite more references

***Response:*** More research published after 2017 has been cited, especially in the rewritten Introduction, to further introduce the trends of research in this field.

**Reviewer 2-2.** Please provide an overview of existing problems and the solutions and suggestions needed to address them in form of a flowchart

***Response:*** A research flowchart has been added to show the whole research process, existing problems and potential solutions in detail.

**Reviewer 2-3.** Lack of a general "Conclusion" section after which suggestions are made

***Response:*** The Conclusion section has been added to summarize all the main results in the three aspects of the *Material and Decay Investigation*, the *Problematic Conservation Cases Analysis*, and the *Conservation Measures Optimization Strategies*.

We are greatly appreciative of your comments on our paper. We look forward to hearing from you.

Thank you and best regards.

Yours sincerely,

Minghao Zhang

407255050@qq.com
